# Monocyte heterogeneity and functions in cancer

**DOI:** 10.1002/JLB.4RI0818-311R

**Published:** 2019-02-18

**Authors:** Claire E. Olingy, Huy Q. Dinh, Catherine C. Hedrick

**Affiliations:** ^1^ La Jolla Institute for Allergy and Immunology La Jolla California USA

**Keywords:** Monocytes, cancer, tumor microenvironment, myeloid cells

## Abstract

Monocytes are innate immune cells of the mononuclear phagocyte system that have emerged as important regulators of cancer development and progression. Our understanding of monocytes has advanced from viewing these cells as a homogenous population to a heterogeneous system of cells that display diverse responses to different stimuli. During cancer, different monocyte subsets perform functions that contribute to both pro‐ and antitumoral immunity, including phagocytosis, secretion of tumoricidal mediators, promotion of angiogenesis, remodeling of the extracellular matrix, recruitment of lymphocytes, and differentiation into tumor‐associated macrophages and dendritic cells. The ability of cancer to evade immune recognition and clearance requires protumoral signals to outweigh ongoing attempts by the host immune system to prevent tumor growth. This review discusses current understanding of monocyte heterogeneity during homeostasis, highlights monocyte functions in cancer progression, and describes monocyte‐targeted therapeutic strategies for cancer treatment.

## Graphical abstract

Review on monocyte heterogeneity, monocyte roles in cancer progression, and therapeutic strategies to target monocytes for cancer treatment.

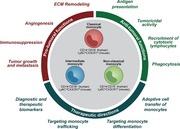

**Abbreviations**
CAR: chimeric Ag receptor
cMoP: common monocyte progenitor
DC: dendritic cell
ECM: extracellular matrix
Flt3: fms related tyrosine kinase 3
GMP: granulocyte‐macrophage progenitor
HIF: hypoxia‐inducible factor
LFA‐1: lymphocyte function‐associated antigen 1
MDP: Mϕ‐DC progenitor
MHCII: MHC class II
M‐MDSC: Monocytic myeloid‐derived suppressor cell
MMP: matrix metalloproteinase
moDC: monocyte‐derived DC
scRNA‐Seq: single cell RNA sequencing
Treg: Regulatory T cell
TAM: tumor‐associated Mϕ
VEGF: vascular endothelial growth factor

Monocytes are mononuclear phagocytes that comprise a major population of innate immune cells that circulate in the bloodstream and traffic to tissues during steady state and at increased rates during inflammation. These cells play key roles in supporting tissue homeostasis, initiating and propagating host responses to pathogens, and resolving immune responses before excessive tissue damage occurs.^1^ Recently, monocytes have emerged as important regulators of cancer development and progression, with different subsets appearing to have opposing roles in enabling tumor growth^2^ and preventing metastatic spread of cancerous cells.^3^ Monocytes also serve as a primary source of long‐lived tumor‐associated Mϕs (TAMs) and dendritic cells (DCs) that shape the tumor microenvironment.^4^ An understanding of monocyte heterogeneity and roles at different stages of cancer progression is critical for improving our understanding of cancer's ability to evade the immune system and ultimately to design novel therapies to enhance antitumoral immunity. In this review, we summarize current work that has explored monocyte heterogeneity and function in the context of cancer and highlight key strategies that leverage monocytes for improving cancer therapies.

## 1 MONOCYTE HETEROGENEITY

Over the last 40 years, our understanding of monocytes has progressed from viewing these cells as a homogenous population to a heterogeneous system of cells that display diverse functions in response to different stimuli. Human monocytes universally express the MHC class II (MHCII) receptor HLA‐DR, integrin αM (CD11b), and CD86, while murine monocytes express CSF1 receptor (CSF1R, CD115), CD11b, and FcγRI (CD64). Currently, 3 subsets of monocytes have been established: classical monocytes (CD14^+^CD16^–^ in human, Ly6C^hi^CD43^lo^CX3CR1^lo^ in mouse), nonclassical monocytes (CD14^lo^CD16^+^ in human, Ly6C^lo^CD43^hi^CX3CR1^hi^ in mouse), and intermediate monocytes (CD14^+^CD16^+^ in human, Ly6C^int^CD43^hi^CX3CR1^hi^ in mouse).^5^ This long‐standing nomenclature was first defined in the late 1980s using 2‐color flow cytometric detection of CD14 and CD16 Ags on human PBMCs.^6^

Whether multiple monocyte subsets were also present in mice remained unclear until the early 2000s. The first evidence that murine monocytes were also composed of distinct subsets emerged in work that identified 2 populations with different patterns of CX3CR1, CCR2, and CD62L expression.^7^ Two years later, a landmark study confirmed the presence of CX3CR1^hi^ and CX3CR1^lo^ monocyte subsets with significant differences in chemokine receptor expression, adhesion molecule profiles, and trafficking within homeostatic and inflamed tissue.^8^ While these initial studies relied on engineered mice with green fluorescent protein inserted into the *Cx3cr1* locus, subsequent studies have identified Ly6C as a more specific marker for discrimination of circulating monocyte subsets and reinforced previously reported phenotypic differences.^9^, ^10^, ^11^ Human monocyte subsets were also found to display differential expression of CX3CR1 that corresponded with similar phenotypes to those observed in mouse, suggesting that mouse and human monocyte subsets may share similarities.^8^ Genome‐wide transcriptome profiling^10^ and adoptive transfer assays to assess patrolling and response to inflammatory stimuli in immunocompromised mice^12^ later reinforced that classical and nonclassical monocytes in mouse and human are counterparts.

Additional surface markers have been useful for improving discrimination of monocyte subsets. We recently used mass cytometry (CyTOF) to demonstrate that inclusion of HLA‐DR, CCR2, CD36, and CD11c markers substantially increases the gating purity of human monocyte subsets, particularly CD14^+^CD16^+^ intermediate monocytes.^13^ Ly6C, MHCII, CD43, and Treml4 have enabled better identification of murine monocyte subsets, particularly within tissues where Ly6Chi monocytes can down‐regulate Ly6C, but maintain low expression of CD43 and Treml4.^14^ High expression of Treml4 is also useful for discrimination of intermediate monocytes from Ly6C^hi^ and Ly6C^lo^ populations.^15^ The numbers of these markers will likely continue to expand as high‐dimensional techniques are used to further profile monocyte transcriptional and proteomic states.

Single‐cell transcriptional profiling has challenged our understanding of heterogeneity within well‐established immune populations and monocytes are no exception. Deep single cell RNA sequencing (scRNA‐Seq) using Smart‐Seq2 recently revealed 2 novel human monocyte subsets.^16^ While most (about 70%) of CD14^+^C16^+^ monocytes typically classified in the intermediate subset fell within classical (Mono1) and nonclassical (Mono2) monocyte clusters, the remaining CD14^+^CD16^+^ cells formed 2 new rare clusters termed Mono3 and Mono4 (12% of total monocytes). Mono3 highly expresses genes associated with cell cycle, differentiation, and trafficking, while Mono4 expresses high levels of cytotoxic gene signatures including NK and T cell activation genes. To date, no additional studies have validated the existence of these novel subsets, developed methodologies to isolate Mono3 and Mono4, or sought to identify their function.

In mice, scRNA‐Seq revealed that monocytes primarily cluster into 4 types, 2 of which overlap with conventionally defined classical Ly6C^hi^ monocytes and nonclassical Ly6C^lo^ monocytes.^11^ The other 2 monocyte clusters were primarily Ly6C^int^ monocytes with 1 subset showing intermediate expression of classical monocyte genes and the other expressing MHCII‐associated genes and *CD209a*. The *CD209a*‐expressing subset is likely similar to the previously identified subset of Ly6C^+^ monocytes that express DC‐related genes, including those encoding CD209a, fms related tyrosine kinase 3 (Flt3, CD135), and MHCII, and give rise to DCs following exposure to GM‐CSF.^17^ Although additional studies are needed to confirm and understand the functional roles of new monocyte subsets, these studies indicate that conventional definitions of monocyte subsets will likely be modified and expanded, particularly within the intermediate subset.

## 2 MONOCYTE DEVELOPMENTAL ORIGINS AND FATES

Understanding the origin and fates of monocytes is crucial to understanding how these cells respond in cancer settings. Monocytes circulate in peripheral blood during steady state after developing from a lineage‐committed bone marrow progenitor, the common monocyte progenitor (cMoP), which was first discovered in mouse.^18^ The human cMoP was recently identified in human umbilical cord blood and bone marrow.^19^ In both mouse and human, cMoPs are unipotent monocyte progenitors that express the stem cell marker CD117, as well as the C‐type lectin CLEC12A and CD64. In contrast to mouse cMoPs, human cMoPs express CD135, a cytokine receptor and early hematopoietic marker.

Monocyte development is regulated by sequential expression of key transcription factors in the following order: PU.1, IRF8, and KLF4, which has been reviewed previously by our group.^20^ PU.1 (encoded by *Spi1*) is a master regulator that is required for development of all myeloid cells and regulates expression of downstream factors such as IRF8 and KLF4.^21^ IRF8 is expressed at low levels in hematopoietic stem cells, but up‐regulated in common myeloid progenitors, granulocyte‐Mϕ progenitors (GMPs), and cMoPs, ultimately restricting differentiation toward mononuclear phagocytes and limiting neutrophil generation.^22^ Many monocyte‐specific genes are directly controlled by IRF8, but IRF8 also induces expression of KLF4, which is required for monocyte development.^23^

The precise relationship of cMoPs to other myeloid developmental lineages such as GMPs and Mϕ‐DC progenitors (MDPs) is not fully understood. GMPs are a heterogeneous population that contains multiple progenitors with various degrees of restriction for differentiation into monocyte, DC, and granulocyte lineages.^24^ Refining the definition of conventional human GMPs to include only CLEC12A^hi^CD64^int^ cells excludes cells with lymphoid or DC potential and identifies a population of cells that sequentially produces cMoPs and monocytes.^19^ However, traditionally defined MDPs (CD115^+^CD116–) phenotypically overlap with both GMPs and cMoPs, indicating that current definitions of progenitor populations are likely heterogeneous. In mice, both MDPs and GMPs can give rise to monocytes, with each appearing able to generate phenotypically different monocytes.^25^ GMP‐derived monocytes possess a subset of monocytes with neutrophil‐like gene expression and MDP‐derived monocytes can express DC‐related genes.^25^ Future work will likely leverage scRNA‐Seq, epigenetic profiling, and more advanced fate mapping strategies to better understand the developmental hierarchy of monocytes and their progenitors.

Following production of cMoPs in the bone marrow, generation of monocyte subsets is generally believed to involve differentiation of cMoPs into classical monocytes and subsequent conversion into nonclassical monocytes in blood circulation, with intermediate monocytes representing a transition state.^9^, ^26^ Early work demonstrated this conversion in mice using in situ bead‐based labeling and clodronate liposome depletion,^9^ but has been confirmed by others using adoptive transfer techniques,^27^ complex fate mapping strategies in transgenic mice,^26^ and epigenetic mapping.^11^ Evidence that this conversion also occurs in humans was recently demonstrated by tracking accumulation of intravascularly administered deuterium‐labeled glucose in healthy individuals and through adoptive transfer of human CD14^+^ monocytes in humanized mice.^28^

The development and survival of nonclassical Ly6C^lo^ monocytes was first found to depend on the orphan nuclear receptor Nr4a1.^29^ Our laboratory demonstrated that Nr4a1‐deficient mice primarily lack nonclassical monocytes and those that are present display abnormal cell cycling, dysfunctional patrolling behavior, and enhanced apoptosis.^29^ Nr4a1 is also expressed in T cells and Mϕs, motivating us to use epigenetic approaches to search for more specific regulatory factors specifically active during Ly6C^lo^ monocyte generation. We identified a super‐enhancer region upstream of the Nr4a1 gene that binds the transcription factor KLF2 and exclusively regulates Ly6C^lo^ monocyte development.^30^ In addition to KLF2, a similar epigenetic approach identified the transcription factor C/EBPβ as a regulator of the Nr4a1 gene and generation of Ly6C^lo^ monocytes.^11^ In agreement with these findings, Ly6C^lo^ monocytes are absent in C/EBPβ‐deficient mice.^31^ Ly6C^lo^ monocyte development may also rely on Notch2 expression and interaction with endothelial cells expressing Notch ligand delta‐like 1 in bone marrow and splenic vascular niches.^32^ Consequently, conversion of Ly6C^hi^ monocytes into Ly6C^lo^ monocytes depends on epigenetic modifications that could be in part regulated by interactions with their surrounding microenvironment.

While most nonclassical monocytes appear to derive from classical monocytes,^11^, ^26^ current work does not rule out the existence of an unidentified lineage‐restricted progenitor that is able to differentiate into nonclassical monocytes without passing through a classical monocyte intermediary. Indeed, a population of segregated‐nucleus‐containing atypical Ly6C^lo^ monocytes that plays key roles in fibrosis develops from a specialized GMP‐dependent progenitor without induction of Ly6C expression.^33^

After differentiation, classical monocytes rely on CCR2 to exit the bone marrow, as well as traffic into tissues and lymph nodes^34^, ^35^ along gradients of CCR2 ligands CCL2, CCL7, and CCL12. CCR2‐deficient mice accumulate Ly6C^hi^ monocytes in bone marrow, but the frequency of circulating Ly6C^lo^ monocytes is minimally affected, indicating that CCR2 is less important for their trafficking.^34^ Entry into lymph nodes requires l‐selectin (CD62L), whose expression is limited to Ly6C^hi^ and Ly6C^int^ monocytes.^35^ Classical monocytes have a half‐life in blood circulation of less than 1 day in humans and mice during steady state.^26^, ^28^ Conversely, nonclassical monocytes display a longer lifespan of 7 days in humans and at least 2 days in mice. Ly6C^lo^ monocytes rely on a unique set of chemokine receptors for trafficking, including CX3CR1 and sphingosine‐1‐phosphate receptor 5.^36^, ^37^ Computational models predict that only 1% of classical monocytes convert into intermediate and subsequently nonclassical monocytes during homeostasis, while the remainder are predicted to extravasate or undergo cell death.^28^ This supports a model where classical monocytes rapidly convert into nonclassical monocytes, traffic to tissues or the lymphatic system, or undergo apoptosis, the latter of which has not been extensively explored.

Monocytes have been detected outside of the circulation, including in skin, lung, and lymph nodes^35^ and human tumors.^38^, ^39^ However, classical monocytes homeostatically entering tissues also give rise to Mϕs and DCs in steady state.^26^, ^40^, ^41^ In mice, most Mϕs appear to be embryonically derived and self‐renew through local proliferation.^26^, ^42^ However, monocytes are also able to repopulate tissue Mϕ populations and self‐renew, adopt similar gene expression, and perform tissue‐specific functions.^42^, ^43^ The mechanisms underlying tissue‐specific differences in monocyte recruitment remain unclear, but they likely depend on environmental cues and tissue accessibility both during homeostasis and inflammation. Classical monocytes are recruited at higher rates to inflamed tissues and are able to attract other immune cells by secreting cytokines and antimicrobial factors.^14^, ^34^ Their differentiation into Mϕs and DCs is regulated by key factors such as CSF1 (M‐CSF), GM‐CSF, and Flt3 ligand^41^ and transcription factors such as IRF4 and MAFB.^44^

A proportion of nonclassical monocytes patrol the vasculature in a lymphocyte function‐associated antigen (LFA)‐1 and CX3CR1‐dependent manner,^36^ although whether nonclassical monocytes that display patrolling activity are transcriptionally distinct from those that do not patrol has not been investigated. Patrolling monocytes scavenge endothelium‐derived cellular debris and flag‐damaged endothelial cells for disposal by recruited neutrophils in a TLR7‐dependent manner.^45^ While nonclassical monocytes primarily remain in the vasculature during homeostasis,^35^ they appear to be able to extravasate during inflammation, although they may do so at lower rates than classical monocytes. Whether nonclassical monocytes differentiate after exiting vasculature remains unclear, although evidence exists that they are able to give rise to Mϕs with an alternatively activated phenotype and display anti‐inflammatory properties in certain contexts.^36^, ^46^, ^47^ The fate of nonclassical monocytes during infection, injury, and disease requires further investigation and is likely dependent on both tissue‐specific cues and type of inflammatory signal.

One of the primary challenges to studying monocyte function in tissues is the limited availability of technologies that discriminate between monocytes and monocyte‐derived cells, including monocyte‐derived Mϕs and DCs. Surface markers such as F4/80, MHCII, and CD11c are up‐regulated once monocytes enter tissue, while Ly6C and CD11b are down‐regulated, but these markers are often expressed along a continuum between blood monocytes and cells in tissue.^40^, ^48^ In vitro studies have revealed that factors such as M‐CSF, LPS, IFN‐γ, and IL‐4 have significant impacts on human monocyte gene expression,^49^ which likely mirrors at least some aspects of in vivo differentiation. Other modifications include metabolic changes and increases in cell size that accompany differentiation into Mϕs or DCs.^14^ Simultaneously monitoring these changes in tissues where the monocyte‐to‐Mϕ transition may not be spatially and temporally synchronized remains difficult. Tools such as depleting Abs (including Gr‐1, CD11b, and CD115 Abs), clodronate liposomes, and genetically engineered mouse models (for example, CCR2‐diptheria toxin receptor, CSF1R‐driven MaFIA, and CX3CR1^–/–^ mice) have provided useful insights into monocyte biology, but are typically unable to selectively target monocytes without having concomitant and direct effects on other mononuclear phagocytes. Interpretation of these tools becomes even more challenging in inflamed tissues, including cancer, where rates of monocyte recruitment and differentiation are higher. One strategy to reduce challenges associated with definitively discriminating monocytes and monocyte‐derived Mϕs/DCs is to refer to all cells with monocytic origins as “monocyte‐derived cells”.^41^, ^50^ This nomenclature would provide unity in the field regarding distinctions between mononuclear phagocyte subsets, particularly in tissues and inflammatory settings. Throughout this review, we will use this nomenclature where appropriate, specifically when evidence is provided that the cells of interest are derived from monocytes, but their precise classification is unclear.

## 3 FUNCTIONS IN CANCER

**Figure 1:**
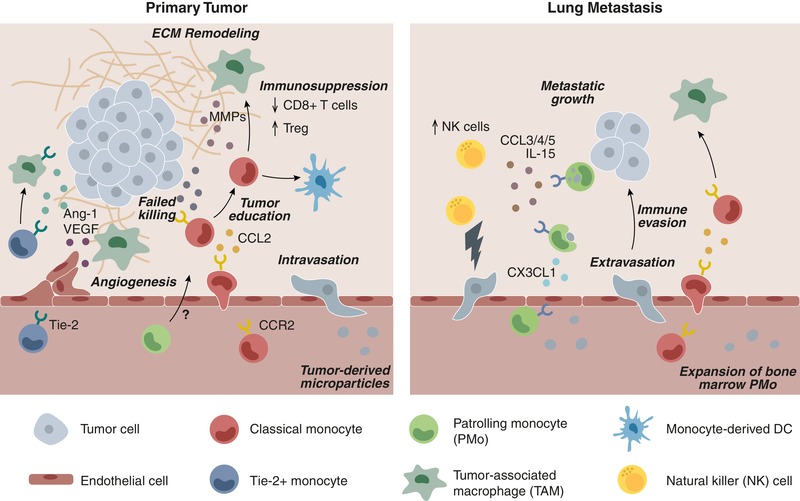
**Recruitment and functions of monocyte subsets to primary tumors and metastatic sites within the lung**. Classical CCR2^+^ monocytes extravasate from the vasculature into primary tumor sites in response to CCL2. Classical monocytes are capable of producing tumoricidal mediators, but are likely reprogrammed within the tumor microenvironment to limit their cytotoxicity. Tumor‐educated monocytes differentiate into TAMs or moDCs. Monocyte‐derived TAMs facilitate tumorigenesis by promoting immune suppression (inhibition of CD8^+^ T cell recruitment/activities and recruitment of Treg), ECM remodeling, angiogenesis, and tumor cell intravasation into the vasculature. Tie‐2^+^ monocytes/Mϕs display pro‐angiogenic functions within primary tumors, although a role for nonclassical patrolling monocytes (PMo) in primary tumors remains unclear. In lung metastatic sites, classical monocytes are recruited in a CCL2‐dependent manner, promote metastatic seeding, and have similar protumoral effects. PMo home to tumor metastases in a CX3CL1/CX3CR1‐dependent manner, where they engulf tumor material and produce chemokines that stimulates recruitment of cytotoxic NK cells. Tumor‐derived microparticles within the vasculature expand bone marrow pools of PMo to increase immune surveillance. Tumor immune evasion within primary and metastatic sites requires protumoral signals to outweigh ongoing attempts by the host immune system to prevent tumor growth

Monocytes can display diverse functions at different stages of tumor growth and progression (summarized in Figure 1). Phenotypically similar monocytes can even appear to perform opposing roles due to differences in cancer type/tissue of origin, subtle differences in tumor microenvironment, stage of tumor growth, and experimental model (summarized in Table 1). Here, we discuss current evidence of the functions that different monocyte subsets can perform in cancer and highlight topics that need further investigation in monocyte cancer biology.

**Table 1:** Pro‐tumoral and antitumoral functions of monocytes and monocyte‐ derived cells in cancer

Subset		Function	Mouse references	Human references
Classical	Protumoral	Differentiation into pro‐tumoral TAMs	PyMT primary mammary tumors and spontaneous lung metastases^2^, ^48^; subQ mammary TS/A tumors^51^; orth. hepatocellular carcinoma^73^; orth. Lewis lung carcinoma^120^; orth. pancreatic ductal adenocarcinoma.^72^, ^76^	Non‐small cell lung cancer.^120^
		Metastatic cell seeding	Lung metastasis of i.v. Met‐1 mammary or B16 melanoma cells^2^, ^3^; liver metastasis of orth. pancreatic ductal adenocarcinoma.^76^	Nude mice bearing orth. mammary xenografts.^2^
		Suppression of T cell function	SubQ mammary TS/A tumors^51^; orth. hepatocellular carcinoma^73^; orth. GM‐CSF‐transduced B16 melanoma^74^; orth. pancreatic ductal adenocarcinoma.^72^, ^76^	Hepatocellular carconima^73^; melanoma^74^; pancreatic cancer.^76^
		Recruitment of Tregs	SubQ RMA‐S lymphoma tumors^78^; orth. pancreatic ductal adenocarcinoma.^76^	
		Angiogenesis	SubQ mammary TS/A tumors^51^; orth. mammary tumors.^125^	
		ECM remodeling	Metastatic lung squamous carcinomas^102^; subQ Lewis lung carcinoma.^103^	Lung squamous carcinoma.^102^
	Antitumoral	Tumor cytotoxicity		In vitro studies of monocytes derived from ovarian cancer^59^ and in culture with multiple cancer cell types.^57^
		Ag presentation	SubQ^82^, ^111^ or i.v. B16 melanoma.^83^	
Non‐classical	Protumoral	Angiogenesis (Tie‐2^+^ monocytes)	SubQ N202 mammary tumors^91^; subQ Lewis lung carcinoma.^93^	Nude mice bearing subQ U87 glioma xenografts^92^; NOD/SCID mice bearing subQ colorectal xenografts.^87^
		Suppression of T cell function	Orth. colorectal tumors.^97^	
	Antitumoral	Tumor cytotoxicity		In vitro studies of monocytes with primary leukemia cells.^60^
		Prevention of metastasis	PyMT mammary tumor spontaneous lung metastases^3^; lung metastasis of i.v. Lewis lung carcinoma^3^ and B16 melanoma.^3^	
		Engulfment of tumor material	Lung metastasis of i.v. Lewis lung carcinoma^3^ and B16 melanoma.^3^, ^64^	
		Recruitment of/correlation with NK cells	Lung metastasis of i.v. B16 melanoma.^3^, ^69^, ^70^	Non‐small cell lung cancer.^38^
		Inhibition of Tregs		Melanoma.^81^

TAM indicates tumor‐associated Mϕ; Tregs indicates regulatory T cells; ECM indicates extracellular matrix; subQ indicates subcutaneous; orth. indicates orthotopic; i.v. indicates intravascular.

### 3.1 Monocyte recruitment to tumors

Monocytes appear to be recruited throughout tumor progression, including during early stages of tumor growth^48^, ^51^ and establishment of distal metastases.^2^, ^3^, ^52^ In multiple models, CCL2 has emerged as the primary mediator of monocyte recruitment. CCL2 expression increases with neoplastic progression in both human and mouse models of colitis‐associated colorectal cancer.^53^ In PyMT mice bearing mammary tumors, classical Ly6C^hi^ monocytes are recruited in a CCL2‐dependent manner to both primary tumors and pulmonary metastases.^2^, ^48^ A similar pattern of recruitment occurred for CD14^+^CD16^–^ monocytes adoptively transferred into nude mice, suggesting that classical monocyte recruitment is a feature of both mouse and human tumors.^2^

Monocytes are likely deployed to primary tumor sites at least in part from the spleen, as splenectomized mice have fewer TAMs and impaired development of lung tumors,^54^ and CCL2 production increases in the spleens of tumor‐bearing mice.^55^ The relative contribution of splenic monocytes remains unclear, as bone marrow monocytes were shown to have a competitive advantage in migration to tumors in adoptive transfer studies and photoconversion of bone marrow and splenic monocyte reservoirs.^56^ These differences could reflect anatomical differences in tumor microenvironment (orthotopic lung tumors vs. subcutaneously implanted lung tumors) or suggest that even low levels of splenic monocyte recruitment can have significant impacts on tumor progression. Additional work is needed to better understand the precise trafficking of monocytes between organs such as bone marrow, blood, and spleen, as well as lymphatics and lymph nodes during tumor progression.

### 3.2 Direct tumoricidal functions

Monocytes appear to have the cellular machinery to directly kill malignant cells by cytokine‐mediated induction of cell death and phagocytosis. Most tumoricidal activity has been demonstrated in vitro, thus whether monocyte‐mediated killing is part of the in vivo antitumoral response during cancer progression needs further exploration. Peripheral blood monocytes exposed to IFN‐γ or IFN‐α produce the protein TRAIL, which is able to induce cell death in TRAIL‐sensitive cancer cells.^57^ However, many cancer cells are resistant to TRAIL‐mediated apoptosis and TRAIL can instead stimulate secretion of protumoral cytokines such as CCL2 and IL‐8.^58^ Monocytes can also induce cancer cell death through Ab‐dependent cellular cytotoxicity, which both CD14^+^ and CD16^+^ monocyte subsets have the capacity for.^59^, ^60^ CD16^+^ monocytes require contact with tumor cells and TNF‐α signaling for induction of tumor cell cytolysis.^60^ Monocytes collected from peripheral blood or ascites fluid of human ovarian cancer patients display reduced capacity for Ab‐dependent cytolysis and phagocytosis of tumor cells upon activation in vitro.^59^ In renal cell carcinoma patients, peripheral blood monocytes secrete factors that promote tumor cell invasion in vitro.^61^ Consequently, malignant cells may be able to coerce monocytes to adopt a phenotype that supports tumorigenesis,^62^, ^63^ thereby overpowering any programmed tumoricidal activities.

Monocytes readily engulf bloodborne tumor‐derived material such as exosomes and large microparticles, indicating that monocyte‐mediated phagocytosis could be an important part of the antitumoral immune response.^3^, ^64^ However, cancerous cells are able to shield themselves from phagocytosis in circulation and within primary tumor sites, for example, through expression of CD47.^65^, ^66^ Circulating monocytes express high levels of SIRPα (CD172a), the ligand for CD47, but CD47/SIRPα interactions have not been extensively explored in monocyte‐mediated phagocytosis of tumor cells. CD47 expression is prognostic for poor clinical outcome in a number of different cancers, including ovarian cancer, glioma, glioblastoma, and non‐small cell lung cancer.^67^, ^68^ Evasion of phagocytosis is likely also critical during metastasis to distal sites, as CD47 expression correlates with the presence of lymph node and distal metastases in non‐small cell lung cancer patients.^68^ Whether CD47/SIRPα interactions play a role in monocyte‐mediated regulation of metastasis (discussed below) also requires further investigation.

The long lifespan and patrolling activities of nonclassical monocytes make them particularly well suited to scavenge tumor cells and debris. In the context of cancer, nonclassical monocytes migrate toward metastatic sites within the lung, where they engulf tumor material and generate cytokines that regulate antitumor immunity.^3^, ^69^ Tumor‐derived exosomes also expand bone marrow pools of patrolling monocytes, which appears to initiate an immune surveillance cascade that prevents metastatic seeding.^70^

### 3.3 Interactions with lymphocytes

Monocytes and monocyte‐derived cells interact with adaptive immunity by directing the recruitment and function of lymphocytes within the tumor microenvironment through paracrine signaling, as well as by serving as Ag‐presenting cells.^14^ Many of the immunosuppressive functions of monocytic cells have been attributed to monocytic myeloid‐derived suppressor cells (M‐MDSCs), which are myeloid‐lineage cells characterized by CD14^+^CD33^+^HLA‐DR^–/lo^ expression (CD11b^+^Gr‐1^+^Ly6C^+^Ly6G^–^ cells in mice) and their ability to suppress T cell function.^71^ Monocytes and M‐MDSCs have many overlapping functions and phenotypic markers, thus whether M‐MDSCs represent a terminally differentiated cell type rather than a cell state induced by cancer and other pathologies requires additional evidence. While many groups have found a separate terminology for immunosuppressive myeloid cells to be useful, we find the MDSC nomenclature to be oversimplifying and not account for the plasticity and heterogeneity that monocytes and their descendants can display. Here, we will discuss the interactions of monocytes and M‐MDSCs with intratumoral lymphocytes in parallel by simply using the terms monocyte and monocyte‐derived cells to facilitate drawing conclusions across studies that may employ different nomenclature.

#### 3.3.1 Lymphocyte recruitment

Monocyte recruitment into tumors appears to be negatively associated with infiltration of cytotoxic CD8^+^ T cells. In renal cell carcinoma patients, circulating monocytes produce higher levels of many inflammatory cytokines and chemokines compared to those in healthy individuals, including TNF‐α, IL‐1β, IL‐6, and CCL3, supporting their likely role in modulating downstream immune responses.^61^ Inhibition of CCR2 or CSF1R prevents monocyte‐derived cell accumulation in murine pancreatic, liver, and melanoma tumors, which is associated with infiltration of more CD8^+^ T cells and reduced tumor growth.^72^, ^73^ Classical monocytes appear to be the primary precursor of immunosuppressive monocyte‐derived cells, at least in melanoma tumors, as CCR2 is required for their accumulation.^74^ CCR2 does not appear to be required for their immunosuppressive function on a per cell basis and instead iNOS and arginase are required for inhibition of tumor‐specific CD8^+^ T cell proliferation.^74^ In further support of CD8^+^ T cells being downstream targets of classical monocytes, CCR2 inhibition has no effect on growth of hepatocellular carcinomas in mice lacking CD8^+^ T cells.^73^ Interestingly, one report found that CCR2‐deficient mice have more tumor‐infiltrating CD4^+^ and CD8^+^ T cells, but no differences in the T cell composition of tumor‐draining lymph nodes.^75^ In these studies, growth of subcutaneously implanted melanoma (B16) and lung (3LL) tumors was not affected by CCR2 deficiency, suggesting that some tumors may rely less on monocytic input for generating antitumoral immunity.

Clinically, intratumoral CCL2 immunostaining negatively correlates with the number of CD8^+^ T cells in patients with hepatocellular carcinoma^73^ and pancreatic cancer patients with tumors expressing high CD8 and low CCL2 display significantly better survival.^76^ Absence of immunosuppressive monocytic cells appears to be important for response to checkpoint immunotherapies, as the frequency of CD11b^+^CD14^+^HLA‐DR^lo^ cells was higher in metastatic melanoma patients who did not respond to anti‐PD‐1 immunotherapy compared to those that did respond and correlated with overall survival.^77^

Within the tumor microenvironment, monocyte‐derived cells can also produce factors such as CCL5 that recruit immunosuppressive regulatory T cells (Tregs).^78^ Monocytes and Tregs are likely involved in a positive feedback loop, as intratumoral Tregs produce factors such as IL‐4, IL‐10, and IL‐13 that direct differentiation of classical monocytes into alternatively activated TAMs with further immunosuppressive functions.^79^, ^80^ Interestingly, nonclassical monocytes are able to induce Fcγ‐dependent cell lysis of Tregs in vitro,^81^ possibly providing a competing effect on Tregs to that of classical monocytes. Whether monocyte‐mediated killing of immunosuppressive T cells occurs in vivo remains to be determined, although melanoma patients responding to anti‐CTLA‐4 checkpoint blockade have more circulating CD14^–^CD16^+^ monocytes and fewer intratumoral Tregs.^81^

In addition to interacting with T cells, multiple reports have demonstrated that nonclassical monocytes are important for recruitment of NK cells to tumors, particularly within metastatic sites.^3^, ^69^, ^70^ Ly6C^lo^ monocytes express high levels of NK chemoattractants such as CCL3, CCL4, and CCL5 and are required for NK cell recruitment to metastatic tumors within lung.^3^ While nonclassical monocytes continue to uptake tumor material in the absence of NK cells, their protective effect against tumor metastasis is lost.^3^ Building upon these findings, IL‐15 was identified as a critical monocyte‐derived signaling molecule required for recruitment of cytotoxic NK cells to metastatic tumor sites.^69^ In human non‐small cell lung cancer tumors, CD16^+^ monocytes are lower in the tumor microenvironment compared to adjacent nonmalignant tissue, which is associated with reduced infiltration of CD16^+^ NK cells.^38^ These findings suggest that the relationship between nonclassical monocytes and NK cells first observed in mouse models of experimental metastasis may be relevant to human solid tumors, although this and interactions with other immune cells requires further investigation.

#### 3.3.2 Ag presentation

Monocytes can differentiate into Mϕs or DCs after entering tissue (discussed below), but they are also able to enter tissues such as skin and draining lymph nodes without differentiation.^35^ Adoptively transferred monocytes up‐regulate MHCII and CD11c after migrating into lymph nodes, which depends on CCR2 and CD62L.^35^, ^82^ Although monocytes appear to have clear Ag‐presenting functions during homeostasis and infection,^14^ their role in presentation of tumor‐derived Ags remains less explored. A population of F4/80^hi^ monocyte‐derived cells that displays similarities to both Mϕs and conventional DCs is able to efficiently cross‐present tumor Ags to CD8^+^ T cells.^83^ These cells may be similar to a previously reported monocyte‐derived DC that enters tumor‐draining lymph nodes, promotes proliferation of CD8^+^ T cells, and is sufficient for generating antitumoral immunity.^82^

While presentation of tumor Ags is generally thought to occur within primary tumor sites or their draining lymph nodes, a recent report demonstrated that intravascular MHCII^+^ monocytes are able to present Ag to CD4^+^ T cells within inflamed glomeruli vasculature.^84^ Whether similar interactions between T cells and monocytes can occur within tumor vasculature or following engulfment of tumor‐derived material in circulation remains an interesting direction for further exploration.

### 3.4 Effects on other components of the tumor microenvironment

Malignant cells are just a small part of the complex ecosystem that makes up a tumor and facilitates its growth. The vasculature, lymphatics, stromal compartment, and extracellular matrix (ECM) composition are nonimmune cells components of the tumor microenvironment that pose unique challenges for delivering therapies and overcoming developed resistance.^4^ Monocytes and monocyte‐derived cells are able to shape many of these microenvironmental features, often in a manner that promotes tumor growth.

#### 3.4.1 Angiogenesis

Induction of angiogenesis is one of the requisite steps in tumor progression, as neoplastic tissues require a vasculature for oxygen and nutrient delivery. Myeloid cells are key regulators of angiogenesis in the tumor microenvironment, including pro‐angiogenic monocytes and TAMs.^85^ Tumors orchestrate the process of forming a new vasculature through low oxygen tension that results in hypoxia‐inducible factor (HIF)‐mediated release of chemokines and growth factors. In glioblastoma tumors, CD11b^+^ myeloid cells are recruited in a HIF‐dependent manner, where they can produce matrix metalloproteinases (MMPs) that release matrix‐bound vascular endothelial growth factor (VEGF) and stimulate tumor angiogenesis.^86^ Peripheral blood CD14^+^ monocytes derived from renal cell carcinoma patients produce more VEGF and better support angiogenesis in vitro compared to healthy monocytes, which requires NK‐κB and IL‐1RA signaling.^61^ Later work proposed specifically that CD16^+^ monocytes (or their progeny) are responsible for tumor VEGF release and this generates a positive feedback loop that recruits additional pro‐angiogenic monocyte‐derived cells.^87^ Whether monocytes can directly support angiogenesis, or first require differentiation and acquisition of Mϕ‐associated functions remains unclear. Consequently, studying monocytes in the context of tumor angiogenesis independently of pro‐angiogenic TAMs faces similar challenges to those discussed in prior sections of this review. For example, treatment with clodronate liposomes results in reduced tumor vascular density in mouse models of metastatic liver cancer and xenografted melanoma, but whether the effect is mediated by depletion of circulating monocytes or TAMs is unclear.^88^, ^89^ Recent evidence suggests that Mϕ differentiation is necessary for supporting vascular function in tumors.^90^

A subset of monocytes express Tie‐2, the receptor for angiopoietin (a vascular growth factor), and are believed to have pro‐angiogenic functions within the tumor microenvironment.^91^, ^92^ In humans, CD16^+^ monocytes contain the primary population of Tie‐2^+^ monocytes, which are capable of promoting vascularization through secretion of factors such as VEGF, TNF‐α, and MMPs and growth of xenografted glioma tumors.^92^, ^93^ The relationship between circulating Tie‐2^+^ monocytes and intratumoral pro‐angiogenic TAMs (including Tie‐2^+^ Mϕs) has not been directly investigated, although Tie‐2^+^ Mϕs also have significant effects on tumor vasculature that enables dissemination of metastatic cancer cells.^94^ The protumoral function of Tie‐2^+^ monocytes/Mϕs appears to extend beyond regulating angiogenesis to include IL‐10‐mediated immune suppression.^95^

Anti‐angiogenic therapies have been successful both in preclinical models and in clinical trials; however, they often fail to produce durable responses.^96^ During treatment with anti‐VEGF therapies, tumors increase production of CX3CL1, which recruit pro‐angiogenic monocyte‐derived cells that drive VEGF resistance.^93^, ^97^ In patients with colorectal cancer, treatment with the anti‐VEGF Ab bevacizumab increases intratumoral and plasma levels of SDF‐1α, a leukocyte migratory factor.^98^ Refractoriness to anti‐VEGF therapy may be prevented by inhibiting monocyte recruitment, which has been demonstrated experimentally using clodronate liposomes and anti‐Gr‐1 Ab.^99^, ^100^ These results indicate that targeting both the vasculature directly and accessory cells such as pro‐angiogenic monocytes/Mϕs is a promising strategy for generating more durable responses to anti‐angiogenic therapies.

#### 3.4.2 Extracellular matrix composition

Similar to other components of the tumor microenvironment, the ECM becomes dysregulated during tumorigenesis, altering cell migration, biomechanics, and signaling.^101^ Classical monocytes are able to remodel the ECM through expression of factor XIIIA, which promotes cross‐linking of fibrin that accumulates in tumor through leaky vasculature.^102^ Densely cross‐linked fibrin provides a scaffold for metastatic tumor cell invasion and is associated with CD14 expression and disease progression in non‐small lung cancer patients.^102^ Mϕs derived from CCR2^+^ monocytes can directly degrade the ECM within tumors by endocytosing deposited collagen.^103^ Additional studies are needed to better understand how monocytes contribute to the composition and organization of tumor ECM, and whether ECM‐derived signals regulate monocyte fate in cancer.

### 3.5 Differentiation into tumor‐associated Mϕs and DCs

Mϕs and conventional DCs display diverse ontogenies, depending on the tissue of origin and pathological context.^26^, ^40^, ^41^ After exiting tumor vasculature, monocytes can differentiate into TAMs or monocyte‐derived DCs (moDCs). As previously discussed, surface markers alone are often insufficient to distinguish monocytes, DCs, and Mϕs, especially in inflamed tissues, thus requiring ontological and functional studies for proper classification.^41^

Classical monocytes are believed to be a major source of TAMs, as they are robustly recruited to primary tumors and metastatic sites, while nonclassical monocytes display much lower levels of recruitment.^2^, ^48^, ^51^ Although CCR2‐deficient monocytes have impaired recruitment into murine breast tumors compared to wild‐type mice,^90^ CCR2‐deficient mice do not display reduced accumulation of TAMs in PyMT breast tumors,^48^ indicating that CCR2 signaling may not be required for TAM accumulation. TAMs can be proliferative, which could provide a compensatory mechanism when monocyte recruitment is impaired.^48^ Recent work has demonstrated that in lung metastatic sites, TAMs are derived from both CCR2‐dependent monocytes and resident interstitial Mϕs, each promoting tumor growth and metastatic spreading, respectively.^104^ Additional work is needed to better understand the relative contributions of monocytes and resident Mϕ populations to TAM accumulation and phenotype across different tumor types.

The precise kinetics of monocyte‐to‐Mϕ differentiation and the transcriptional changes that accompany this transition in the tumor microenvironment remain incompletely understood. In primary breast tumors, recruited CCR2^+^ classical monocytes up‐regulate TAM markers such as F4/80, CD11c, MHCII, and V‐CAM1 within 5 days of arriving to the tumor.^48^ Subsequent Mϕ differentiation relies on adhesion signals such as those generated during CD11b interactions with CD90‐expressing tumor cells.^105^ In the metastatic lung, a population of Ly6C^hi^CD11b^hi^ cells derived from classical monocytes that appear within 18 h appears to serve as precursors for mature TAMs.^52^ Though these cells are morphologically and transcriptionally distinct from both blood monocytes and TAMs, they also appear to be heterogeneous, therefore requiring further investigation. There may be an important spatiotemporal component to monocyte differentiation, as TAMs derived from recently arriving monocytes are more frequently found in collagenous stromal tumor regions and later found in perivascular regions, where they regulate vascular permeability and tumor cell intravasation.^90^, ^106^ Taken together, these results suggest that the differentiation trajectory of recruited classical monocytes is complex, depending on spatial and temporal heterogeneity within the tumor microenvironment.

Monocyte‐derived TAMs are generally believed to be protumoral, enabling both primary tumor growth and seeding of metastatic tumor cells.^2^, ^48^ TAMs appear to be phenotypically and functionally different from Mϕs found in healthy tissues,^48^ shifting the tumor immune microenvironment to support immune evasion, angiogenesis, and metastatic outgrowth. However, this may be more complex and depend on factors such as spatial positioning within the tumor. For example, a higher density of Mϕs at the tumor front in colon cancer patients was associated with a higher density of lymphocytes and improved survival.^107^ For discussion of Mϕ functions within the tumor microenvironment, we refer the reader to excellent reviews elsewhere.^108^, ^109^

DCs make up a minor population of tumor‐infiltrating myeloid cells, with moDCs representing an even smaller fraction.^4^, ^82^, ^110^ Most DCs are derived from lineage‐restricted progenitors that depend on the transcription factors BATF3 and IRF8 or IRF4 for development into convention DC1 or DC2, respectively.^110^ However, DCs can also develop from monocytic precursors, particularly within inflamed tissues such as tumors. moDCs may be characterized as DCs rather than monocytes if they up‐regulate markers such as CD11c and MHCII, adopt dendrite‐like morphology, perform DC‐associated functions such as Ag presentation, and are demonstrated in ontological studies to have monocytic origin.^41^

Monocytes that differentiate into DCs utilize CCR2 to migrate into both tumors and tumor‐draining lymph nodes.^82^ Loss of DCs in CD11c‐DTR mice prevents antitumoral immunity that can be restored by monocyte adoptive transfer.^82^ BATF3 knockout mice bearing B16F10 melanoma tumors lack cross‐presenting CD103^+^Ly6C^+^ cells and are poorly responsive to chemotherapy, but tumor growth can be significantly delayed through adoptive transfer of cMoPs.^111^ Interestingly, in tumor‐bearing CCR2‐deficient mice, Ag loading is redistributed from TAMs to CD103^+^ conventional DCs and more CD8^+^ T cells are recruited.^64^ This finding highlights an additional effect of tumor‐mediated monocyte recruitment in which Ag is diverted to cells that are less effective at generating antitumoral immune response.^64^

The cues that govern monocyte fate decisions between remaining a monocyte, differentiation into a Mϕ or DC, or undergoing programmed cell‐death remain are not fully understood. In a recent study of human breast cancer intratumoral heterogeneity using scRNA‐Seq, the authors inferred a monocytic activation gene signature that reflected a trajectory from blood monocytes to intratumoral monocytes, and from that to other myeloid compartments such as DCs and TAMs.^39^ Similar types of analyses in both mouse models and human cancers will be critical for improving our understanding of monocyte fates within tumors.

## 4 TARGETING OF MONOCYTES FOR CANCER DIAGNOSTICS AND THERAPY

### 4.1 Monocytes as cancer diagnostics

Early diagnosis remains one of the primary challenges in oncology, with most tumors diagnosed at Stage III or IV when therapies are often less effective promoting tumor regression. Current work seeks to identify novel biomarkers that enable earlier cancer diagnosis or improved patient treatment, with peripheral blood representing a desirable sample site due to its ease of access. The absolute frequency of monocytes in peripheral blood is associated with survival in B cell lymphoma^112^ and locally advanced cervical cancer.^113^ More commonly, the ratio of lymphocytes to monocytes has emerged as a prognostic factor, including for B cell lymphoma,^114^ colorectal cancer,^115^ lung cancer,^116^ and ovarian cancer.^117^ For example, in patients with stage III colon cancer, a higher lymphocyte to monocyte ratio was associated with increased time to recurrence and overall survival.^115^ In colorectal cancer patients, peripheral blood monocytes display a gene signature that is not present in healthy monocytes or other types of cancer, highlighting the potential for cancer‐specific biomarkers based on monocyte signatures.^63^

Discrimination of monocyte subsets may provide additional diagnostic power and biological insights given the divergent roles of different monocyte subsets in cancer progression. A lower frequency of peripheral blood CD14^+^CD16^–^ monocytes was associated with their increase in bone marrow and greater survival in pancreatic cancer,^76^ suggesting that pancreatic tumors systemically alter monocyte trafficking. In addition, high CCL2 expression within pancreatic tumors is associated with reduced infiltration of CD8^+^ T cells and poor survival. Similarly, the amount of CCR2 expression in prostate tumors correlates with clinical pathologic stages and is higher in metastatic tissues than the primary tumors from which they derive.^118^

In patients with stage IV melanoma, response to anti‐CTLA‐4 immune checkpoint blockade (ipilimumab) can be predicted by the frequency of peripheral blood CD14^lo^CD16^+^ monocytes.^81^ In a separate study, the frequency of CD11b^+^CD14^+^HLA‐DR^lo^ monocytic cells was lower in metastatic melanoma patients who responded to anti‐PD‐1 immunotherapy compared to those who did not respond.^77^ Using high‐dimensional mass cytometry data, recent work demonstrated that the frequency of classical CD14^+^CD16^–^CD33^+^HLA‐DR^+^ monocytes is higher in melanoma patients responding to anti‐PD‐1 compared to nonresponders.^119^ Collectively, these results indicate that peripheral blood monocytes can contain information about likelihood of responding to immunotherapies, but additional studies are needed to understand which monocyte subsets are the most robust at predicting response to specific therapies.

### 4.2 Targeting monocyte trafficking

Egress of classical monocytes from bone marrow into peripheral blood is mediated by CCR2 signaling, which is also important for recruitment to tumor sites. Blockade of CCR2 signaling with Abs or small molecules targeting either CCR2 or CCL2 reduces tumor growth or metastasis in mouse models of breast,^2^ lung,^120^ pancreatic,^72^, ^76^ liver,^73^ and prostate^121^ cancers. Nanoparticles loaded with CCR2 short interfering RNA, a gene silencing tool, have also been effective at reducing accumulation of monocyte‐derived cells and tumor growth in a mouse model of lymphoma.^122^ While inhibition of CCR2 signaling directly blocks monocyte recruitment, downstream effects include fewer monocyte‐derived TAMs,^73^ impaired tumor angiogenesis,^122^ increased infiltration of cytotoxic T cells,^73^ and prevention of tumor cell metastasis.^2^ Importantly, targeting CCR2 signaling may be synergistic with existing chemotherapy agents, as CCL2 inhibition in combination with docetaxel was more effective at promoting regression of murine prostate tumors than docetaxel alone.^121^

mAbs targeting CCL2 appear to be well tolerated and cause minimal adverse side effects in human patients.^123^ Surprisingly, no improvement in clinical outcome was observed in prostate cancer patients treated with a monoclonal anti‐CCL2 Ab, although the Ab may have been ineffective at neutralizing CCL2.^123^ A recent clinical trial demonstrated the safety of a small molecule CCR2 antagonist in advanced pancreatic cancer patients,^124^ which represents a promising alternative to Ab‐based therapies. Caution is likely needed in clinical translation of CCL2/CCR2‐targeted therapies, as discontinuation of anti‐CCL2 therapy enhances metastasis in mouse experimental models of breast cancer.^125^ Blocking pro‐tumorigenic cues derived from monocyte‐derived cells such as IL‐6 and VEGF may be one strategy to prevent adverse effects caused by termination of anti‐CCL2 therapy.^125^

While CCR2 signaling is the key chemokine receptor for recruitment of classical monocytes, CX3CR1 plays a key role in migration of nonclassical monocytes.^8^ Recruitment of nonclassical monocytes to primary colorectal tumors and metastatic lung tumors is dependent on CX3CR1^3^, ^97^; however, whether CX3CR1 is protumoral or antitumoral remains unclear. Loss of CX3CR1 has no effect on growth of subcutaneously implanted Lewis lung carcinoma tumors,^97^ but significantly reduces tumor growth after orthotopic implantation.^120^ In experimental metastasis models in which B16 melanoma is intravascularly administered, extravasation from the vasculature, homing toward tumor cells, and tumor uptake by nonclassical monocytes is impaired in CX3CR1‐deficient mice.^3^ CX3CR1‐deficient mice display greater tumor burden in this model and fewer intratumoral NK cells, further supporting a nonclassical monocyte‐NK cells axis in tumors.^126^ In patients, high tumor expression of CX3CL1 is associated with infiltration of CD8^+^ T cells and NK cells, as well as better clinical prognosis in gastric and breast cancer patients.^127^, ^128^ These results indicate that CX3CR1 may be a promising therapeutic strategy, but additional understanding of CX3CR1 in the context of cancer is needed and targeting this pathway will likely be challenging.

The SDF‐1α/CXCR4 pathway may function in a similar manner to CX3CL1/CX3R1 signaling, although CXCR4 is more broadly expressed among hematopoietic cells.^86^, ^129^ Inhibition of CXCR4 with the small molecule AMD3100 has improved the efficacy of anti‐angiogenic therapies, purportedly through blocking recruitment of pro‐angiogenic monocytes.^129^, ^130^

### 4.3 Targeting monocyte differentiation

CSF1 (M‐CSF) is a key regulator of monocyte recruitment and differentiation into TAMs,^131^ making CSF1/CSF1R signaling a potential therapeutic target for cancer immunotherapies. A number of different strategies have been used to target CSF1 signaling in monocytes and Mϕs, including mAbs, small molecules, and RNA‐based approaches.^132^ CSF1 antisense oligonucleotide or small interfering RNAs improved survival in mice bearing human mammary tumor xenografts, which was associated with reduced Mϕ infiltration and tumor vascularization.^133^ Abs against CSF1 or CSF1R have been effective at impairing tumor growth in mouse models of pancreatic cancer,^72^ breast cancer,^134^ and glioma,^135^ among others. Inhibition of CSF1R or CSF1 typically results in reduced accumulation of TAMs, impaired vascularization, and enhanced infiltration of cytotoxic CD8^+^ T cells.^134^, ^136^ Therefore, it is perhaps unsurprising that blocking CSF1R enhances antitumor T cell responses and promotes regression of pancreatic tumors and melanoma during checkpoint blockade.^137^, ^138^ CSF1R blockade has also demonstrated efficacy in enhancing response to clinically used chemotherapy agents such as paclitaxel^134^ or gemcitabine.^72^

Translation of CSF1R‐targeted therapies to the clinic have been minimal,^132^ with demonstration of efficacy limited to a Phase I clinical trial in which the CSF1R mAb RG7155 suppressed growth of diffuse‐type giant cell tumors.^136^ This may be in part due to reservations about long‐term efficacy of CSF1R inhibitors. Following resection of subcutaneously implanted Lewis lung carcinoma tumors, BLZ945 (a small molecule CSF1R inhibitor) treatment resulted in a surprising increase in lung metastases, likely due to inhibition of NK cell recruitment to the tumor.^139^ While BLZ945 improves survival in a mouse model of glioma, over half of gliomas ultimately develop resistance to CSF1R inhibition.^140^ Recent work demonstrated that the efficacy of CSF1R inhibitors may be limited by a concomitant increase in recruitment of protumoral immunosuppressive neutrophils, which can be prevented by co‐administration of a CXCR2 inhibitor.^141^ These studies highlight potential obstacles facing clinical translation of CSF1R inhibitors, while demonstrating that combination therapies can help overcome these limitations to improve long‐term outcomes.

### 4.4 Additional therapeutic directions

The divergent role of monocyte subsets in cancer progression is perhaps best exemplified by adoptive transfer studies. Intravascular adoptive transfer of sorted Ly6C^lo^ monocytes into Nr4a1‐deficient mice that lack Ly6C^lo^ monocytes reduces tumor metastasis by nearly 4‐fold in an experimental model of melanoma metastasis to the lung.^3^ Conversely, adoptive transfer of Ly6C^hi^ monocytes nearly doubles tumor metastasis, consistent with their protumoral functions. While transfer of antitumoral monocyte subsets represents a promising cancer therapeutic strategy, significant challenges remain to ensure adequate numbers of patient‐derived monocytes that retain their in vivo functions can be obtained. Unlike chimeric Ag receptor (CAR) T cells that have been successful in the clinic for treatment of leukemia and lymphomas,^142^ monocytes display limited proliferative capacity ex vivo and can be a challenging target for gene editing. Despite these challenges, a Phase I clinical trial was recently initiated to explore autologous transfer of monocytes collected by apheresis to patients with recurrent chemotherapy‐resistant ovarian cancer.^143^ Results from this clinical trial will be informative for future directions in clinical monocyte transfer therapies.

Targeting monocytes may be also a promising strategy to mitigate adverse events in patients being treated with existing cancer therapies. For example, in a humanized mouse model of leukemia, monocytes are the primary source of IL‐1 and IL‐6 released during the onset of cytokine release syndrome following CAR T cell therapy.^144^ Depletion of monocytes with CD44‐directed CAR T cells or clodronate liposomes prevented cytokine release syndrome.^144^ The efficacy of CAR T cell therapies can also be limited by concomitant induction of immunosuppressive myeloid cells in some cancers such as sarcomas.^145^ Co‐administration of all‐trans retinoic acid reduces the abundance of immunosuppressive monocytes in blood and enables antitumor immunity by sarcoma‐targeted CAR T cells.^145^ Radiation is a major source of toxicity for cancer patients treated with radiation therapy and bone marrow‐derived monocytes and Mϕs are critical for regeneration of the nervous system after radiation‐induced damage.^146^ Administration of G‐CSF to mice with focal‐brain irradiation injuries increases infiltration of monocytes/Mϕs and improves functional neural repair.^146^ These examples highlight the potential value of monocyte‐directed adjuvant therapies for improving cancer treatment.

## 5 FUTURE DIRECTIONS

Although many important functions have been associated with monocytes in the progression of cancer, the cues that regulate their fate and differentiation into protumoral or antitumoral cells remain incompletely understood. For example, classical monocytes primarily differentiate into pathogenic TAMs, yet they can also differentiate into moDCs that are required for effective adaptive immune responses. Patrolling monocytes potently prevent tumor metastasis within the lung, yet they also appear able to support angiogenesis within primary tumors. Whether these functions are entirely governed by microenvironmental cues within tumors or preprogrammed in specific monocyte subsets remains unclear. A better understanding of how to shift the balance toward monocyte fates that aid in antitumoral immunity will be critical for the design of more effective immunotherapies.

## DISCLOSURES

The authors declare no conflict of interest.
